# Mentoring in crisis does not need to put mentorship in crisis: Realigning expectations

**DOI:** 10.1017/cts.2020.508

**Published:** 2020-07-10

**Authors:** Kenzie A. Cameron, Lauren A. Daniels, Emily Traw, Richard McGee

**Affiliations:** 1 Division of General Internal Medicine and Geriatrics, Department of Medicine, Northwestern University Feinberg School of Medicine, Chicago, IL, USA; 2 Northwestern University Clinical and Translational Sciences Institute, Northwestern University Feinberg School of Medicine, Chicago, IL, USA; 3 Faculty Affairs, Northwestern University Feinberg School of Medicine, Chicago, IL, USA

**Keywords:** Mentorship, expectations, realignment, academia, COVID-19, crisis

Mentoring is a critical component in academia, described as both a responsibility of faculty and a catalyst for career success for junior faculty.^1^ Healy and Welchert’s definition of mentoring highlights reciprocity between mentor and protégé.^2^ The need for intentional reciprocity dramatically expands as we fight the COVID-19 pandemic and are forced to confront our country’s sustained mistreatment of minoritized populations, underscored by the inescapable truths of multiple recent deaths of Black and Brown citizens. Sustaining mentoring relationships during these times requires reciprocal recognition, validation, and acknowledgement of our experiences and realignment of our expectations.

## Recognize the World in Crisis

We face a world in crisis and confront uncertainty daily; to avoid discussion of crises is to ignore the lived experiences of others and ourselves. We must identify how these crises affect our own perspectives, productivity, priorities, mental states, and working conditions. Tuning in to our perspectives allows us to identify our own personal and professional needs. By recognizing changes in our own working environments, we will be more open to understanding changes that others face, providing us the opportunity to jointly identify and overcome these unexpected challenges.

## Validate Shifting Professional and Personal Roles and Responsibilities

Academic roles have shifted. Many with clinical expertise were called in for extra shifts, served on newly created ethics committees, and worked with health systems to rapidly launch telemedicine to care for patients. Researchers without clinical expertise faced huge challenges with lost and disrupted studies. For all of us, day-to-day experiences changed and we had to reprioritize how we spend our time.

Some faculty received messaging suggesting this unexpected time at home provided an opportunity for increased writing productivity.^3^ As others have noted, increased productivity is not feasible for all;^4^ not all have the luxury of converting time previously spent in the lab or running experiments into uninterrupted writing time. Quite the opposite scenario played out for many, particularly caregivers, and specifically women.^3,5,6^ Parents of babies and toddlers wished for extra hands to hold their children; parents of school-aged children expanded their roles of parent, teacher, and professional. Those caring for spouses, siblings, and parents may have done so alone, unable to access community programs, adult day care, or in-home care. Almost everyone experienced increased email and virtual meetings for contingency planning, readjusting recruitment strategies, and identifying next steps for research currently on-hold. It is not hard to understand how many may simply be attempting to *maintain* productivity, not needing the additional stress of an expectation to *increase* productivity.

## Acknowledge Grief and Practice Compassion

Mentorship in a time of crises necessitates acknowledging grief.^7^ Grief may arise from a death or non-death loss, that is, a “living loss.”^8^ As a result, people feel powerful mental, emotional, and physical reactions.^8,9^ The COVID-19 pandemic has resulted in death and a multitude of non-death losses: loss of in-person connections, routine, economic security, freedom, a sense of certainty, and normalcy. The losses felt by individuals, groups, and society as a whole due to the ongoing violence against Black and Brown people, exemplified by the killing of George Floyd, are immense. Yet, it is hard to know, and often harder to understand, the depth of grief others feel.

At times like these, compassion for ourselves and others can facilitate interactions. Compassion for oneself could include accepting your video conferences may involve unplanned guest appearances by children, pets, and family; avoiding admonishing yourself for not completing your to-do list; acknowledging feelings of grief and loss; and remembering to stand up and stretch. Compassion in mentoring may include reaffirming common mentor and mentee responsibilities: listening carefully to each other’s goals and expectations, identifying areas of strength and growth and discussing how they may have changed, and confirming flexibility and willingness to alter expectations and change plans.^10^

## Realign Mentorship Expectations

As we begin a slow return to re-engaging in person in our workspaces, in a world undeniably different from when we left, remember that alignment of expectations in a mentorship relationship was never meant to be a one-time, static event, but rather an iterative, living process.^11–13^ Mentors and mentees share this responsibility; yet we believe that mentors, due to their leadership role, need to set the tone and take the first steps:

*Acknowledge the importance of and need for reassessing goals*. Mentors need to be receptive to the reality that timelines will be altered and mentees’ goals may change.
*Make time and space to engage in conversations with your mentees*. Even as our institutions move toward “opening,” work hours are being staggered. Many mentors and mentees will remain working from home for an undetermined time. We have lost both the informality and frequency of quick check-ins in the hall, the elevator, or before a meeting. Mentors should carefully consider how and when you will and can mentor your mentees. Through this process, mentors need to acknowledge their own limitations, whether they relate to privacy, family commitments, or their own mental and physical health.
*Start the conversation with your mentee, then listen carefully*. Minimize your mentee’s fear of raising difficult, perhaps even controversial, topics by raising them yourself. The stresses of the time may require engagement in new conversations that delve into identifying how personal and professional differences influence the expectations either of you hold.^13^
*Be open and honest with your mentee*. Share your own challenges, to reinforce that the crises we face affect all of us. You may not fully understand the extent of a mentee’s grief, or their trauma, but accept the difficulties they face and be honest that you want to listen and learn.
*Be aware of the cultural differences and similarities between yourself and your mentees*. Identify and learn how your own culturally shaped beliefs, perceptions, and judgments inform your mentoring practices and relationships.^14^

## Conclusion

As our world has changed, so too may our mentoring relationships. Interactions may change in frequency or duration, the channels by which we communicate, the products we complete, and the expectations we hold. Recommitment to a continual process of realigning expectations avoids placing mentorship itself in crisis.
